# Physiology and Murder

**Published:** 1888-10-06

**Authors:** 


### The Hospital

A Weekly Institutional Journal of

**Science, Medicine, Nursing, and Philanthropy.**

*For Hospitals, Asylums, Medical Practitioners, Students, Nurses, Families, Parishes, Congregations, and the Charitable Public.*

Vol. V. No. 106] [October 6, 1888.

### Physiology and Murder.

The theory started by the coroner that Annie Chapman and the other Whitechapel victims were murdered by a semiscientific hand, with some kind of scientific end in view, is still believed in many quarters. Can it by any possibility be true? It is stated that an American, presumably a physician, is about to publish a book on a medical speciality, and, to make his arguments and demonstrations complete, determines to issue with his book, not drawings or diagrams like commonplace physiologists, but the actual organ which is the subject of his prelections. The man of science requires large numbers of specimens, perhaps hundreds or even thousands, for his purpose is to issue one with each copy of his book. Where are they to come from? They can only be furnished by the living or the dead—by the living at the imminent risk of life, by the dead at the cost of desecration as wanton as it is sad.

Assuming that this is the fact, the American seems to have been conscious that his object was one which presented great difficulties, perhaps impossibilities. But he was a scientific enthusiast; one who had probably rid himself completely of the ignorant prejudices and contemptible weaknesses which ordinary people dignify with the names of "respect for the living" and "reverence for the dead." What to him were either living or dead compared with the advance and propagation of scientific knowledge? Man is simply an animal, a mammal at the top of a series; an animal with certain peculiarities, of which science, for purposes of correct classification, must take careful account. Of what significance are a few individual members of the class compared with the value of full and accurate knowledge of the type? Even poetry, which may be presumed to be often completely antipodean to science, recognises this. Does not our noblest bard proclaim that—

> The individual withers,
> But the age is more and more?

Nature herself is invoked in aid of this manifest and unquestionable dictum, and she is complimented by her scientific patrons with approving enthusiasm because—

> So careful of the type she seems,
> So careless of the single life.

It is all very strange, very admirable, very full of hope and promise for the future, very humane, very gentle, and has not the faintest suspicion of cold-bloodedness, of moral imbecility, of murder, or of devilishness about it.

The scientific American wanted his specimens, and probably thought he would have no difficulty in procuring them in the Great Republic. There must be many degraded women in the vast cities of the Western world, and not a few active and adventurous spirits among the men who are fully abreast of the scientific intelligence of the times. There seems to have been at least one skilled operator if the story of the Texas murders is to be believed. These latter would naturally be in full sympathy with the physiologist, and quite willing to collect him as many specimens as he wanted—for a consideration. The specimens need not cost more apiece than those African butterflies which missionaries so often collect for enthusiasts, to their own comfort and profit. Even if it were found difficult to obtain what was wanted among the white populations, there are plenty of negresses in America who might conveniently furnish the organs required. If still the demand were too large, it might be possible to make a raid on one or two tribes of Indians, provided the price per organ were sufficient. In all cases where animals are hunted and slain for their skins, or their feathers, or their livers, or, as in the latest instance, for another organ, the work of hunting can only be undertaken as a business when it "pays." The scientific American, being a man of science and an American, of course knows the value of the "almighty dollar," and he is prepared to make woman-hunting profitable. Although the offer of £20 for each human uterus brought to him may have been a piece of "brag," there seems no reason to doubt that that was the sum named.

It must have come upon this distinguished pioneer of science with a shock of surprise and disgust when he found how stupid and apathetic the Americans were on the great subject of scientific progress. Still more must he have been astonished and disgusted when his offers of £20 per specimen failed to procure for him what he required. With the zeal of an indignant apostle he appears to have shaken off the dust of his feet against the stars and stripes, and resolved to seek the accomplishment of his noble and humane object in Europe. Perhaps he tried Berlin, or still more probably Paris, or most likely of all Vienna. That we do not know at present, but it may be hoped we shall know in due time. It is surely impossible that so burning and shining a light of science should be permitted to remain in obscurity. Failing all these places of scientific light and leading, there still remained as a *dernier ressort* the great metropolis of Britain, the "hub of the universe," the centre of all that is most brilliant in science, all that is most dogmatic, all that is most "cocksure," all that is most free from prejudice of every kind, insular, religious, sentimental—London, the capital of the world. To London accordingly the human creature, who is surely fast reverting, not to the simian but to the crocodilian type, directed his steps. He sought a pathological museum—where it is not stated—expounded his necessities to the curator, and proposed his terms of reward.

It is not said that the curator had any thoughts of shooting him like a noxious beast, or even of handing him over to the police. It does, however, appear that he distinctly informed him there was no probability of securing what was wanted by means of the museum. What was to be done next? We do not know at present what steps may have suggested themselves to the mind of the undaunted man of science. What we do know is that he is said to have offered £20 each for certain female human organs. Who knows how far and wide he made his offer known among private persons connected with museums? Who knows how many attendant Fausts of post-mortem rooms have had dark and sinister interviews with this physiological Mephistopheles? Imagination shrinks from picturing him hurrying from place to place with the price of blood in his hands, tempting the ill-paid attendants to murder the living or to desecrate the poor and friendless dead. What we do know is that women have been murdered; that one of them at least has been mutilated as no one could have mutilated her who had not had knowledge and experience of operations upon the dead; that others have been the subjects of similar mutilation; that a "shabby-genteel" and foreign-looking man was seen in the neighbourhood of the murders for some time, and that he cannot now be discovered.

Is this, then, the length to which physiologists have come in their determination to make their knowledge full and accurate? We do not say that Annie Chapman was murdered by a physiological Burke or Hare. What we affirm, and that on the sober word of a London coroner, is that the sum of £20 each has been offered either in boast or in earnest, at one or more pathological museums for certain organs of the female human body. Following upon this offer, one woman has been murdered and that organ removed; and other women have been slain and similarly mutilated. Last week we expressed the conviction that the man who should suggest this explanation of these terrible crimes must be a fool. This week we have to confess that human nature is more cruel, more fiendish, more blind and mad than we had thought it possible. For what useful service could the organ of the dead woman render to any mortal man? It could teach him practically nothing which is not known already. At every pathological museum specimens of the same organ, which have been obtained by the most legitimate means, may be seen and examined, though they may not be purchased and taken away. There are animals by the score which have similar organs, and which can be obtained for less than £20 each. But that will not do. This passion for a minute, but often entirely useless exactness possesses the minds of certain scientific men; and they attach more importance to a newly-discovered nerve centre of the hundredth part of a pea in magnitude than they would to the fall of an empire or the rise of a religious faith. It is measureless stupidity—it is blindness, it is madness. Where is their sense of proportion, where their understanding of man, his life and his destiny as a whole? These mole-like scientists so obstinately live in their own narrow underground domains that they do not seem even to be aware of the vast worlds of fact and experience which lie beyond their ken. Science let us have by all means; Knowledge, and evermore knowledge. But at least let those who seek it have some kind of measures and scales by which to estimate its value. The mind of this imbecile American physiologist is like that of a man who would be willing that every town in his country should be burnt down in order to ascertain the shape of the buttons which adorn the mayor's coat.

